# Dempster–Shafer Theory for Modeling and Treating Uncertainty in IoT Applications Based on Complex Event Processing

**DOI:** 10.3390/s21051863

**Published:** 2021-03-07

**Authors:** Eduardo Devidson Costa Bezerra, Ariel Soares Teles, Luciano Reis Coutinho, Francisco José da Silva e Silva

**Affiliations:** 1 Laboratory of Intelligent Distributed Systems (LSDi), Federal University of Maranhão, São Luís 65080-805, Maranhão, Brazil; ariel.teles@ifma.edu.br (A.S.T.); luciano.rc@ufma.br (L.R.C.); fssilva@lsdi.ufma.br (F.J.d.S.eS.); 2 Federal Institute of Maranhão, Araioses 65570-000, Maranhão, Brazil

**Keywords:** Internet of Things, uncertainty, complex event processing, Dempster–Shafer theory

## Abstract

The Internet of Things (IoT) has emerged from the proliferation of mobile devices and objects connected, resulting in the acquisition of periodic event flows from different devices and sensors. However, such sensors and devices can be faulty or affected by failures, have poor calibration, and produce inaccurate data and uncertain event flows in IoT applications. A prominent technique for analyzing event flows is Complex Event Processing (CEP). Uncertainty in CEP is usually observed in primitive events (i.e., sensor readings) and rules that derive complex events (i.e., high-level situations). In this paper, we investigate the identification and treatment of uncertainty in CEP-based IoT applications. We propose the *DST-CEP*, an approach that uses the Dempster–Shafer Theory to treat uncertainties. By using this theory, our solution can combine unreliable sensor data in conflicting situations and detect correct results. *DST-CEP* has an architectural model for treating uncertainty in events and its propagation to processing rules. We describe a case study using the proposed approach in a multi-sensor fire outbreak detection system. We submit our solution to experiments with a real sensor dataset, and evaluate it using well-known performance metrics. The solution achieves promising results regarding Accuracy, Precision, Recall, F-measure, and ROC Curve, even when combining conflicting sensor readings. *DST-CEP* demonstrated to be suitable and flexible to deal with uncertainty.

## 1. Introduction

The Internet of Things (IoT) paradigm has emerged from the proliferation of objects and mobile devices (i.e., “things”) connected, which results in the acquisition of periodic event flows from different devices and sensors that need to be processed [1]. IoT proposes to expand the current structure of the Internet into a network of interconnected objects that not only collect information from the environment but also interact with the physical world to provide services such as information transfer and analysis, applications, and communications [2,3]. Some examples of IoT applications are in Smart Cities, Smart Factories, Smart Buildings, Smart Homes, and Smart Cars [4]. Understanding these IoT environments and their interactions are becoming an increasingly difficult task [3]. Thinking in such interactions as event notifications to be processed should improve the analysis and interpretation of information.

Events can be conceived as unique occurrences of interest in time. However, recognizing patterns that comprise a particular composition of these occurrences to obtain meaningful information for systems also makes the processing of these events complex [5]. In recent years, event-driven communication and processing models have been widely disseminated, studied by the academic community, and accepted by the industry [6,7]. Several application domains have benefited from the event processing area involving both event-based processing and communication.

In event processing, the key idea is to explore temporality and causality relationships between events to recognize patterns promptly. This type of processing can reveal opportunities or threats while they arise, so executing decisions in a constrained time. Therefore, systems capable of efficiently processing data to recognize complex situations of interest immediately when they occur define Complex Event Processing (CEP) [5]. CEP can also be interpreted as a programming paradigm that supports reactions to event data flows in real time through a set of methods and techniques to perform such processing [8,9]. Identifying near real-time occurrences of interest in the face of a wide variety of event flows from various sources is one of the essential requirements in many event processing applications [10].

Event processing has become common in many IoT applications. When considering event processing, such applications assume that event flows are stable, and events are captured while they happen. Furthermore, such applications assume that all communication channels and sensors are reliable, and event processing does not occur on uncertain data [11,12]. However, in real-world IoT applications, event producers usually generate event flows that are unreliable. In this case, such applications have to operate in real physical environments, which are intrinsically complex and unpredictable, with data acquired from devices and sensors that can have different levels of precision defined by different manufacturers. In addition, in these applications, the information included in event flows may contain imprecision at the origin of the event [13]. For example, sensors and devices that emit events may be defective or affected by failures, have poor calibration, and therefore produce inaccurate data, which is then considered uncertainty at the event source [14].

The common and widely used techniques for analyzing event flows from sensors, devices, and systems present in IoT is CEP [15]. By providing a structure based on a set of rules, CEP enables to extract information about patterns of relationships between simple events (i.e., primitive event) and derived complex events [16] (also called “situation” [17]). Uncertainty in CEP is normally observed in events (e.g., data or sensor readings) or in rules that may propagate unreliable results or conflicting conclusions. Sources that produce events transmitted to the CEP may incorporate inaccuracies [13,18] into the event content [12,19,20,21,22] or even provide unreliable judgment about a derived event occurrence [23,24].

Imprecise data propagated to the event flow processing can result in erroneous information or incorrect system behavior. The event flow in sensor networks may be noisy and may not be assumed to be valid and stable. Several studies have been performed on the processing of noisy event flows [25,26,27] and complex events processing under uncertainty [6,12]. According to Akila et al. [15], uncertainty under primitive events significantly degrades the precision of results in terms of false positives and false negatives. Consequently, CEP does not meet expectations of achieving objectives and decision-making capabilities in mission-critical applications under uncertainty conditions.

The problem of data uncertainty in IoT applications (i.e., by considering limited precision of sensors) causes the second problem of propagating uncertainty from primitive events to the inference of derived events (i.e., complex events). By recognizing these problems, the scope of this paper comprises the treatment of uncertainty on event data from primitive events that directly affect CEP rules by resulting in uncertainty propagation to derived events in IoT applications.

This study proposes an approach to model and treat uncertainties in event flow processing from sensors and devices in IoT applications. We investigate a mathematical theory capable of modeling uncertainties observed in CEP, representing and calculating the propagation of uncertainty inherent in complex events. From this investigation, we take advantage of the Dempster–Shafer Theory (DST) [28,29], which is flexible and useful for modeling and treating uncertainties. DST enables the propagation of uncertainty values from sensor and device information and indicates certainty about CEP results. We developed our approach, named *DST-CEP*, originally founded in DST elements, specifically designed to model and treat uncertainty in CEP-based IoT applications.

This paper is organized as follows. Section 2 presents preliminaries to understand the proposed solution. Section 3 discusses related works, while Section 4 introduces the *DST-CEP* approach for IoT applications. Section 5 presents a case study conducted with the *DST-CEP* approach. Section 6 presents the experimental evaluation performed with the proposed solution, and discusses results and limitations. In the end, conclusions concerning our study and some directions for future work are drawn in Section 7.

## 2. Background

### 2.1. Complex Event Processing in IoT

In 2001, Luckham [16] defined events as immutable records of occurrence of an action or state change, and event processing as a method of tracking and analyzing information flows (i.e., data) about things that happen (i.e., events), and derive conclusions. Event processing presents an event as something that happens (or labeled as “happening”) and that, according to Luckham [5], is related to the processing of occurrences in real time, i.e., with events that are just happening [30,31].

Event processing provides real-time visibility of a wide variety of event data. However, a key feature of events is that they cannot be fully predicted [32]. Although it is not possible to predict a critical event, detecting an event of interest is an essential requirement in many event processing applications [10]. Events can be conceived as unique occurrences of interest in time, but being able to identify patterns/situations that comprise a particular composition of these occurrences to obtain meaningful information for systems makes the processing of these events complex. Therefore, systems capable of efficiently processing data to recognize complex situations of interest immediately when they occur define CEP. Additionally, CEP can be seen as a programming paradigm that supports reactions to real-time event flows through a set of methods and techniques to perform such processing [5,8,9].

**Figure 1:**
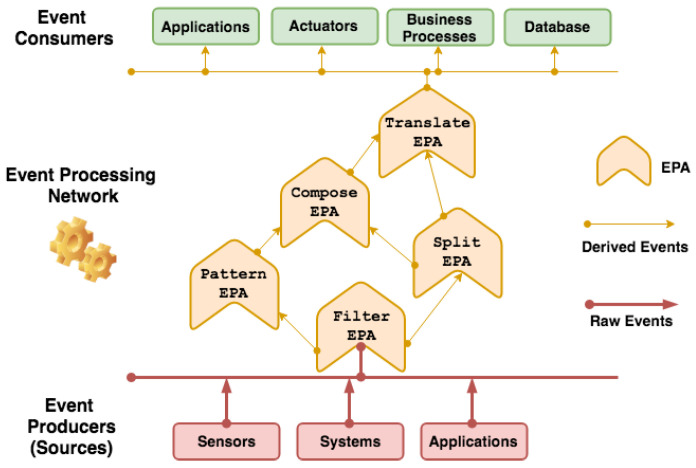
Event Processing Network (EPN) overview.

In IoT, there is a multitude of information flows, computer-based human collaborations, electronic businesses, and interactions with software agents that continuously generate a large amount of data. Platforms developed for IoT demand a massive amount of interaction flows that indicate some aspects of Big Data, such as volume, speed, variety, and veracity (or especially uncertainty) of data [8,11]. IoT applications have benefited from using event processing structures to consume information flows from various sources and derive situations. An example is the structure of the Event Processing Network (EPN) presented in Figure 1, where events are created by producers, which are entities (e.g., sensors and client applications) that generate occurrences of interest to IoT applications.

The event flow is the result of an event sequence created and sent by producers. CEP workflow continuously processes this sequence of input events, and analyzes and manipulates it. Next, there are derived events that are delivered to consumer entities (e.g., monitoring applications). These events generally represent notifications about detected situations [33]. More precisely, it is possible to identify event hierarchies in which each query is executed by an intermediate stage of CEP processing, known as Event Processing Agent (EPA). From the communication between EPAs, through the connection between their input and output terminals, there is an EPN [30].

### 2.2. Dempster–Shafer Theory

The Dempster–Shafer Theory was formally introduced by Glenn Shafer [29] based on the extension of the work by Arthur Dempster [28] and also presented as “Evidence Theory” by dealing with evidence-based hypothesis support. Pieces of evidence can be seen as events that occurred or can occur in a system. The relationship between evidence and hypothesis corresponds to a cause and consequence relationship, i.e., evidence implies a hypothesis or a set of hypotheses. The strength of an evidenced-hypothesis assignment, or the strength of that implication, may be quantified by the declaration of a person (i.e., a specialist), study, organization, or entity (data source) that provides information for a scenario [34]. According to Stephens [35], it is possible to believe in a hypothesis if it agrees with a perception. However, there may exist a distance between perceptions and reality that makes the notion of evidence, which may be strong or weak, about a specific hypothesis. According to Shafer [29], it is not expected that there is an objective relationship between an evidence and a given hypothesis that determines a precise numerical degree. Instead, by having assumed perceptions and understandings that constitute a body of evidence, a number may be announced, which represents the degree to which the evidence is believed to support a given hypothesis, hence the degree of belief that is desired to be attributed to that hypothesis.

The Dempster–Shafer Theory initially assumes a Frame of Discernment (FoD), which is a set of primitive hypotheses (e.g., $h_{1}$ and $h_{2}$) about some problem domain or environment. FoD is represented by Θ and consists of a set of elements of the environment of interest $\Theta =\{h_{1},h_{2}\}$. All subsets formed by the disjunction of Θ elements give rise to $2^{\Theta }=\left\{Ø,\left\{h_{1}\right\},\left\{h_{2}\right\},\left\{h_{1},h_{2}\right\}\right\}$ possible hypotheses (i.e., four hypotheses). The relevance of belief for each element of $2^{\Theta }$ (or each hypothesis $h_{i},\;\ldots ,h_{n}$) is represented by a function called mass function (m). The mass function is the initial source (or basic attribution) of the DST that indicates how strong a piece of evidence supports a hypothesis. Based on the evidence, the DST associates a number in the range from 0 to 1 that measures how much the evidence should agree with a hypothesis. The mass function does the mapping between attributed beliefs and hypotheses.

By definition, the DST requires that the sum of the masses attributed to the hypothesis of interest to be equal to one. Formally, if $h_{i}$ represents any element of $2^{\Theta }$, then $m:2^{\Theta }\;\to \;\left[0,1\right]$ is satisfied according to Equation (1).

(1) $$\begin{array}{c} m(Ø)=0\\ \sum_{h_{i}\in 2^{\Theta }}m\left(h_{i}\right)=1 \end{array}$$

By considering that additional pieces of evidence become available, it is possible to combine them to produce a better estimate about hypotheses. The Dempster Combination Rule combines masses to produce a new mass that represents a consensus of original and possibly conflicting pieces of evidence. For example, if multiple sources provide new notifications or pieces of evidence about the hypothesis on the frame of discernment, they may register distinct mass values about the same or different hypotheses. Therefore, the combination rule enables to calculate new beliefs based on the combination of different mass values.

Given the two different mass attributions $m_{1}$ and $m_{2}$, the combination rule makes the orthogonal sum of these masses indicated by the notation $m_{1}⊕m_{2}$. Formally, the orthogonal sum (⊕) is defined in Equation (2).

(2) $$m_{1}⊕m_{2}\left(A\right)=\sum_{B\cap C=A}m_{1}\left(B\right)\;\cdot \;m_{2}\left(C\right)$$

A difficulty with this calculation is that it can assign masses to some elements with a null intersection. However, by definition, the mass assigned to the empty set is equal to zero (m(Ø)=0). To deal with this case, masses assigned to all other sets are normalized as follows defined in Equation (3).

(3) $$m_{1}⊕m_{2}\left(A\right)=\frac{1}{k}\;\cdot \;\sum_{B\cap C=A}m_{1}\left(B\right)\;\cdot \;m_{2}\left(C\right)$$

The new normalized mass value attributed to *A* is then the orthogonal sum of the mass values divided by the normalization factor *k*, which is defined in Equation (4).

(4) $$k=1-\sum_{B\cap C=Ø}m_{1}\left(B\right)\;\cdot \;m_{2}\left(C\right)$$

The DST may be employed in the process of analyzing uncertainties present in IoT scenarios, so considering the processing of unreliable sensor data. The DST can be seen as a fundamental feature for analyzing complex events from sensor data (evidence) and defining the most assertive hypothesis within the Θ domain.

## 3. Related Work

The method of how uncertainty is treated in CEP-based IoT applications depends on the uncertainties and specificities related to the problem domain. Dealing with all possible uncertainty cases in CEP in a single method seems improbable, so that each related work focuses on its particular restrictions. Consequently, the literature offers a wide variety of approaches, not only technical but also from a conceptual perspective [8,15,36]. In this section, we initially present the first models proposed in the literature to deal with uncertainty in event processing. Next, studies whose approaches process probabilistic (or uncertain) events are presented. We discuss approaches that treat many types of uncertainties (e.g., in attributes, rules, propagation). In addition, we explore solutions applied to specific domains focusing on the diversity of approaches and methods.

### 3.1. Approaches Based on Probabilistic Events and Bayesian Solutions

Wasserkrug et al. [37] described the first model proposed to deal with uncertainty in event processing, which was extended in [6,7,12]. The initial study of Wasserkrug et al. [37] proposed a formal representation of events and composition of events, so enabling inference of complex events even in uncertainty scenarios. Two types of uncertainty were considered. The first one was the uncertainty caused by the imprecision of information signaled by the source of the event (e.g., defective or imprecise sensors). The second type of uncertainty considered is inherent in relationships between events. This study was based on the probability theory to represent the two associated uncertainties and quantify the probability of the events derived. In addition, a Bayesian Network (BN) was built while events were occurring. In this step of the solution, the objective was to calculate probabilities of event occurrences based on the probabilities defined by the BN.

Another study by Wasserkrug et al. [7] aimed to provide a mechanism for reasoning about uncertain events. The first focus of the study was to provide efficiency in deriving events under uncertainty considering a large number of incoming events. The second focus was on the probabilities associated with events that should be correctly captured and represented. A sampling-based algorithm was suggested as a mechanism for approximating derived event probabilities.

Cugola et al. [19] proposed another solution to deal with uncertainty in CEP. The authors highlighted the inability of CEP to consider, model, and propagate uncertainty. The solution called CEP2U assumes that the different attribute values are independent and models uncertainty derived from the rules using probability theory and BN to dependent sources. CEP2U automatically translates a rule into a corresponding BN, which is refined and enriched by a domain specialist. Therefore, the BN is updated with relevant information that can influence the occurrence of events in the network and avoid wrong deductions. Finally, the probability of the derived events is computed considering the probabilities and expert knowledge entered in the network nodes.

### 3.2. Solutions for Specific IoT Application Domains

The study by Artikis and colleagues [13] pointed to the need to address uncertainties in event processing and proposed a classification of possible uncertainty sources. The authors also recognized the need to model and propagate uncertainty, and proposed to use the probability theory as a mathematical basis for performing such tasks. The authors highlighted uncertainties in event processing systems based on previous studies [38,39], and some uncertainty sources presented are: incomplete event flows, erroneous event recognition, inconsistent event notes, and inaccurate event patterns. The study by Artikis et al. [13] was applied to a crime detection scenario, specifically to the modeling of a visual surveillance system able to detect and track people under a wide variety of environmental conditions.

Jarraya et al. [40] proposed the Fuzzy Semantic Complex Event Processing (FSCEP), a model of reasoning and representation of events, which integrates knowledge domain and fuzzy logic. This model aimed to deal with multiple dimensions of uncertainty in sensor data, specifically in a scenario where a person lives in a smart home equipped with several sensors. The uncertainty treated is decomposed into multiple dimensions: freshness, precision, and contradiction. The solution proposed a trust index assigned to each sensor (assigned by the domain specialist) whose values are used to calculate the index for complex events. In the FSCEP model, a simple event is enriched with information from sensor and domain in a process called event semantization. The proposed fuzzification process enables multiple interpretations of the generated fuzzy value to solve contradictions and ambiguities.

Rincé et al. [41] proposed a method for estimating the probability of recognizing a complex event in a short period from a stream of uncertain low-level events. The method addressed uncertainty in the event considering that event detection is not guaranteed (i.e., events may be lost or incorrectly detected). The solution has a model based on a chronicle formalism of events that describes a complex event as sequential composition of simple events. It uses a set of operators based on timestamps and duration intervals of the events. The calculation of the estimated probability of a complex event is done using techniques based on the Markov Chain.

Ma et al. proposed in [42] a solution for modeling events and reasoning from multiple information sources. It integrates domain knowledge and the DST to deal with uncertainty and incomplete information in event flows. Previously, the first study of the authors emerged in [43] from a demand for closed-circuit television bus surveillance systems to detect threats, prevent terrorist attacks, and vandalism. In [44], the solution had advances, and in [45] the DST was used to deal with video analysis and process results of algorithms to classify gender of people.

### 3.3. Our Contribution

The scope of this study is considered a relevant research problem in the literature [8,11,12,13,18,19,46]. Various theoretical methods were explored such as probabilistic approaches, Bayesian, Fuzzy, among others [6,19,40]. However, in the literature, the DST [28,29] is flexible and effective for modeling uncertainty, and widely applied to several areas such as information fusion [47], pattern recognition [45,48,49], decision making [50,51,52,53], selection of suppliers [54,55], optimization problems [56], risk analysis [57,58], and failure diagnosis [59,60]. We presented an overview of the main studies developed to treat uncertainties in CEP, and could observe that uncertainty in CEP is addressed in different ways. However, dealing with inaccurate, incomplete information, uncertainty in events, and propagation of uncertainty using a CEP approach enriched with the DST is a novelty. This is the contribution of our proposed solution. The DST can contribute positively to problems caused by uncertainty in sensor data and promote CEP processing mechanisms that combine multiple sources of divergent or conflicting information in IoT applications.

The approach proposed in this study implements the DST elements to treat uncertainty from multiples sensors in IoT applications. The approach initially assumes a frame of discernment with primitive hypotheses. Evidence about hypotheses arise from sensor reading events. These pieces of evidence are inputs to a set of CEP rules that implement mass functions with a discount factor in uncertain sensors and, subsequently building hypothesis conjectures. From this set of derived hypotheses, the Dempster combination rule is applied to high-level CEP rules to minimize uncertainties and calculate the most plausible hypothesis.

## 4. DST-CEP Approach for IoT Applications

This section describes the *DST-CEP* approach. We first present its event representation and architectural model [61], and how we incorporate them into IoT applications. We then describe *DST-CEP* modeling from a perspective of Dempster–Shafer functions.

### 4.1. Event Representation

One of the resources provided by our solution is a mechanism for representing uncertainty information, so making it explicit in events. The uncertainty modeling in events from the DST functions (i.e., mass function and discount factor) requires a formal representation of the event ($e_{i}$) in the *DST-CEP* approach, as follows in Equation (5) and explained below:

(5) $$e_{i}=\left(Id,Ts,\left\{\left(epl_{i},df_{i}\right),\;\ldots \left(epl_{n},df_{n}\right)\right\},\left\{\left(h_{i},m_{i}\right),\;\ldots \left(h_{n},m_{n}\right)\right\}\right)$$

- *Id*: identification of the production source of events;
- *Ts*: timestamp of the event;
- $epl_{i}$: evidence payload (e.g., location, area, temperature, CO level, smoke level);
- $df_{i}$: discount factor;
- $h_{i}$: hypothesis;
- $m_{i}$: mass value for hypothesis.

Notifications of incoming events in the *DST-CEP* approach are characterized by a level of uncertainty related to the event source (e.g., sensor readings) that needs to be identified, modeled, and calculated as explained in the following sections.

### 4.2. Architectural Model

**Figure 2:**
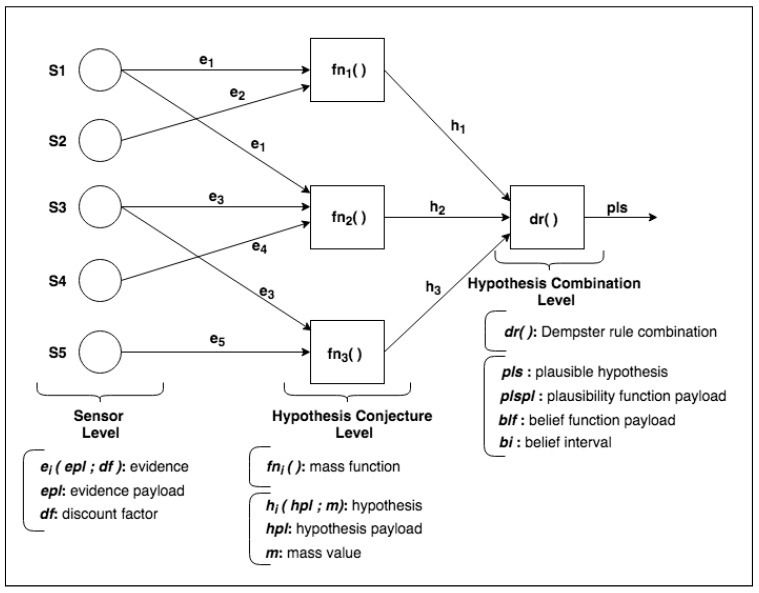
Dempster–Shafer Theory-Complex Event Processing (*DST-CEP*) building block (DSTBB).

We designed the *DST-CEP* architectural model implemented using building blocks. The model includes DST components for processing events originated from a collection of sensors. Figure 2 presents an instance of a *DST-CEP* building block.

Initially, a *DST-CEP* building block (DSTBB) is divided into processing levels. In Figure 2, the Sensor Level has a collection of sensors $S_{1},S_{2},\;\ldots ,S_{n}$ that sends primitive events $e_{1},e_{2},\;\ldots ,e_{n}$ (i.e., sensor readings), which are pieces of evidence as inputs in the *DST-CEP*. Each event $e_{i}\left(epl,df\right)$ is composed of evidence data, called evidence payload (epl), and discount factor (df) calculated from the sensor precision.

The Hypothesis Conjecture Level (Figure 2) has a set of CEP rules that processes primitive events. Results are derived events that represent hypothesis conjectures ($h_{i}$) with the associated mass value *m* for each hypothesis. The generation of hypotheses can occur from the evidence of one or more sensors. The mass value generated for each hypothesis ($h_{1}$, $h_{2}$, $h_{3}$) is calculated through the mass function $fn_{i}\left(\right)$, whose formulation depends on the application domain or expert knowledge. Figure 2 illustrates different mass functions ($fn_{1}\left(\right),fn_{2}\left(\right),fn_{3}\left(\right)$), which represent a set of specific rules defined by one or more specialists.

**Figure 3:**
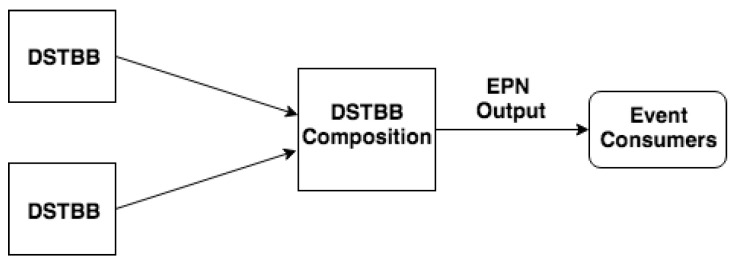
EPN building block.

In the Hypothesis Combination Level, from a set of derived hypotheses, the Dempster combination rule dr() is employed to calculate the most plausible hypothesis (pls). Optionally, another information can compose results, such as the belief function, plausibility function and belief interval. The composition of a set of DSTBBs represents an EPN (or EPN building block), as illustrated in Figure 3.

### 4.3. Modeling Uncertainty in Events

#### 4.3.1. Mass Function

The Dempster–Shafer Theory defines that each hypothesis in a frame of discernment receives a mass value. In *DST-CEP*, we propose to use Algorithm 1 of the mass function adapted from the Min-Max normalization technique [62,63] to calculate the mass value in the range from 0 to 1.
**Algorithm 1:** Mass Function.
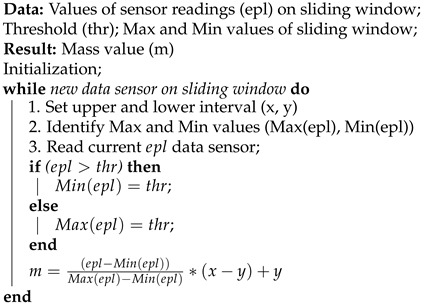


In *DST-CEP*, the mass function consists of directly capturing the evidence payload (epl) and comparing how much it approximates or exceeds a threshold (thr) specific to each sensor. The Max and Min values are extracted from a sliding window [64] containing the latest ten events. Algorithm 1 transforms a sensor reading value (epl) to a mass value (*m*) that fits in the range [x−y], so scaling it appropriately from the threshold (thr) distance based on parameters Min(epl) and Max(epl), which are identified on the current sliding window.

#### 4.3.2. Discount Factor

The *DST-CEP* approach models uncertainty in events by initially incorporating a discount factor (df) for event attributes. A lower discount factor expresses less uncertainty. The discount factor considers the accuracy in sensor readings, measurement techniques, or accuracy levels defined by manufacturers (e.g., ±3% of accuracy, ±0.03 of error). The discount factor is assumed to be known and provided by the sources. For example, a sensor that “knows” its error estimation attaches it to event notifications produced. Alternatively, a domain specialist can provide the discount factor and integrate it into event notifications before processing them.

#### 4.3.3. Mass Value with Discount

The *DST-CEP* approach processes all primitive events received by multiple sensors. Since sensor data are inherently uncertain due to several factors, the mass value related to the events should be discounted by considering imprecision of sensors represented by the discount factor. The reliability of the production source of events can be expressed inversely to the discount factor. The higher reliability (*r*) corresponds to a lower discount factor (df), this means r=1−df. Therefore, the mass value assigned to the production source of events is given with the discount based on the DST, as follows:

(6) $$\begin{array}{c} m^{df}\left(h\right)=\left\{\begin{array}{c} (1-df)\;\cdot \;m(h),h\subset \Theta \\ df+(1-df)\;\cdot \;m(\Theta ),h=\Theta \end{array}\right. \end{array}$$

where 0≤df≤1, which implies:

- the source is absolutely reliable when (df=0);
- the source is reliable with a discount factor df when (0<df<1);
- the source is completely unreliable when (df=1).

Therefore, $m^{df}\left(h\right)$ is the mass value with discount (df), whose result is assigned to *h* representing an element in a frame of discernment.

### 4.4. Modeling Uncertainty in CEP Rules

**Figure 4:**
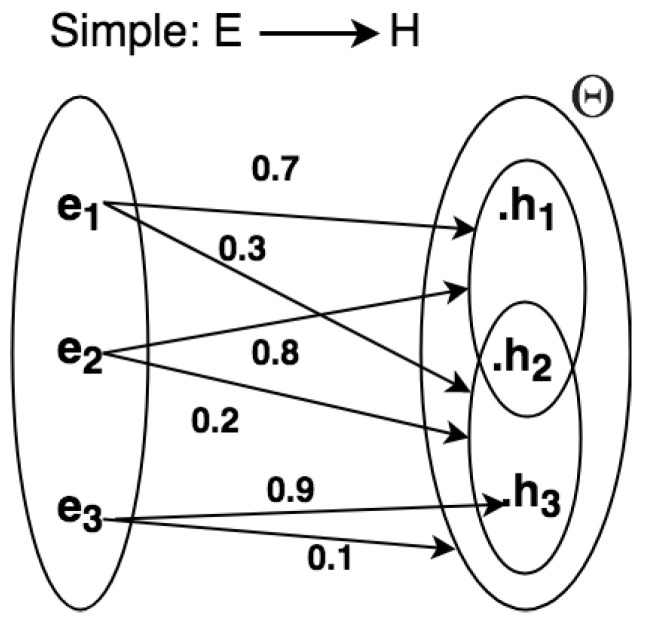
Graph for representing uncertain simple relations.

We present methods to represent uncertainty in CEP rule descriptions using graphs and semi-graphs (by semi-graphs means that both graphs and uncertain relation statements are used together for representation) [65,66,67] for a given domain problem. In Figure 4, the frame of discernment $\Theta =\;\{h_{1},h_{2},h_{3}\}$ and relations can be represented as graphs, so consisting of nodes connected by directed edges. By using graphs, it is possible to add, in a simple manner, uncertain relations directly between the evidential space $E=\{e_{j},\;\ldots ,e_{m}\}$ and the hypothesis space $H=\{h_{i},\;\ldots ,h_{n}\}$.

**Figure 5:**
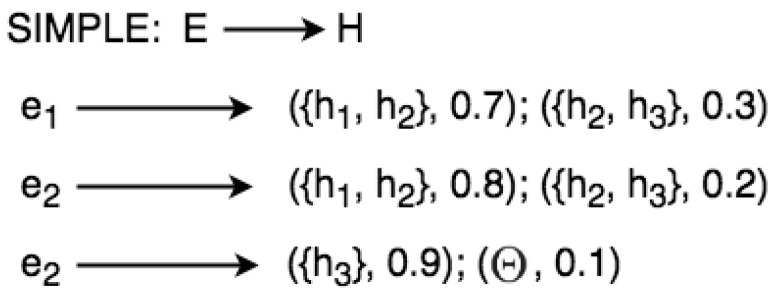
Semi-graph for representing simple rules.

Sometimes, it is difficult to clearly describe uncertain relations of a complex situation in the graph representation. Therefore, a semi-graph is used to represent rules, as shown in Figure 5,

The word SIMPLE in Figure 5 means that the evidential space consists of only one variable. Given the example in Figure 4, we can define a set of rules from the frame of discernment:

- IF $e_{1}$ THEN $\left\{h_{1},h_{2}\right\}$ with 0.7
  $\left\{h_{2},h_{3}\right\}$ with 0.3;
- IF $e_{2}$ THEN $\left\{h_{1},h_{2}\right\}$ with 0.8
  $\left\{h_{2},h_{3}\right\}$ with 0.2;
- IF $e_{3}$ THEN $\left\{h_{3}\right\}$ with 0.9
  Θ with 0.1;

where $E=\{e_{1},e_{2},e_{3}\}$ and $H=\{h_{1},h_{2},h_{3}\}$.

**Figure 6:**
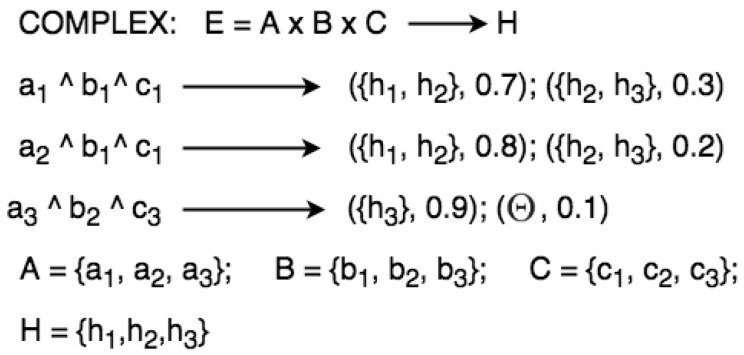
Semi-graph for representing complex rules.

Eventually, when the evidential space has more than one variable, this means that it is COMPLEX. Formally, if the evidential space E is made of A, B, C, and $e_{1}=a_{1}\wedge b_{1}\wedge c_{1}$, $e_{2}=a_{2}\wedge b_{1}\wedge c_{1}$, $e_{3}=a_{3}\wedge b_{2}\wedge c_{3}$, then the variable frames A, B, C of E need to be specified in a semi-graph as illustrated in Figure 6.

**Figure 7:**
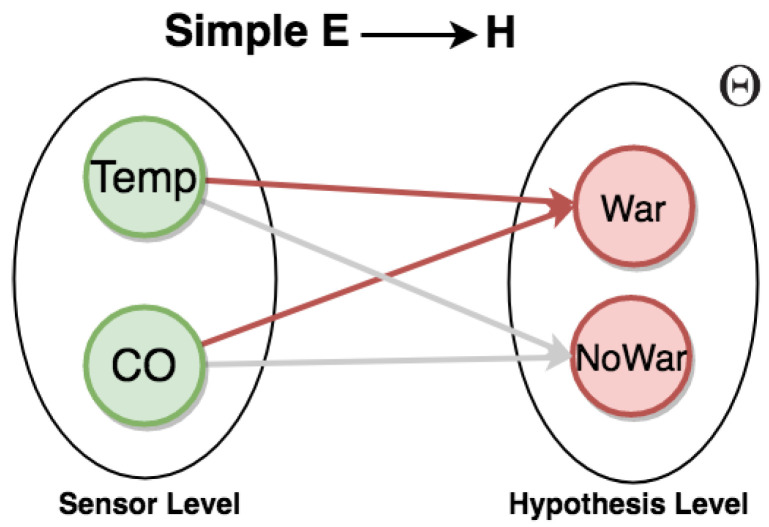
Graph for representing warning events.

In practice, suppose an example of an IoT application. Consider a neighborhood with smart homes, each one equipped with sensors. In particular, sensors record absolute temperature values (*Temp*) of the house, and carbon monoxide (*CO*) sensors analyze the amount of CO gas in the air. Additionally, we can have a set of rules for a frame of discernment (see Figure 7).

For example, to generate the event warning (*War*), the composition of rules follows:

- high CO event occurs when a given carbon monoxide sensor displays a reading with a value above 6000 units;
- high temperature event occurs when a given temperature sensor displays a reading with a value above 45 °C;
- when both high CO and high temperature events occur within 1 min, we have the event warning.

According to the above description, the CEP rules are described in Codes 1, 2, and 3 using the Event Processing Language (EPL) [64].

Code 1: CEP rule HighCO.

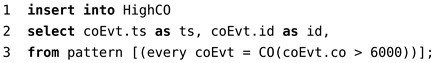



Code 2: CEP rule HighTemp.

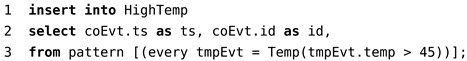



Code 3: CEP rule Warning.

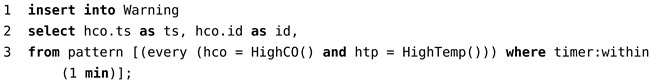



**Figure 8:**
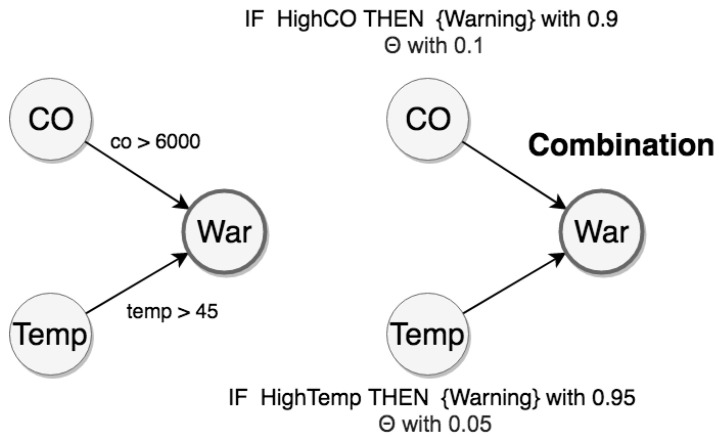
Representations of the warning rule.

To illustrate these CEP rules, we have the respective graphs with statements about uncertain relations in Figure 8.

The evidential space is made of *E = {HighCO, HighTemp}*, and the hypothesis space is made of *H = {Warning, NoWarning, *Θ*}*. From this evidential space, we can use the Dempster combination rule to calculate mass values about the hypothesis of warning events. The adjustment of the mass distribution used to reflect uncertainty of the sources is discussed in Section 4.3 and Section 5.2. Therefore, we can propagate these distributions from the evidence space to the hypothesis space using the Dempster combination rule (Equations (2)–(4)).

## 5. Case Study

This section presents a case study conducted with the *DST-CEP* approach in an IoT application. The case study is explained to didactically present the use of the proposed approach. Moreover, the same case study is also used in the experimental evaluation. Specifically, we present an application that detects fire outbreak in real time based on sensors.

### 5.1. Application Scenario

Consider a multi-sensor fire outbreak detection system that can be used in IoT applications such as Smart Buildings, Smart Homes, or Smart Factories. This system consists of sensors and event processing agents. Sensors are automatic components of the fire detection system, which include flame, smoke, and temperature sensors. They are able to quickly detect physical and chemical information generated by a fire, and transmit them to the processing agents to detect fire. These event processing agents are called detectors, since they use sensor-based information to detect fire (or non-fire).

When that system uses a single sensor to collect information, it might become unreliable due to interference caused by dust, electromagnetism, water vapor, air, light, vibration, and other environmental conditions. Eventually, the system cannot effectively distinguish between early fire signals and ambient interference signals. Therefore, it cannot promptly send fire warnings. In this application scenario, the fire detection system use three sensors to improve the reliability of fire and non-fire detections. Therefore, dealing with uncertainty and processing information collected from multiple unreliable (and possibly conflicting) sensors becomes a major issue.

### 5.2. Processing Levels

The fire detection system structure follows the *DST-CEP* architecture model (Figure 2) and has three processing levels: sensor, hypothesis conjecture, and hypothesis combination.

**Figure 9:**
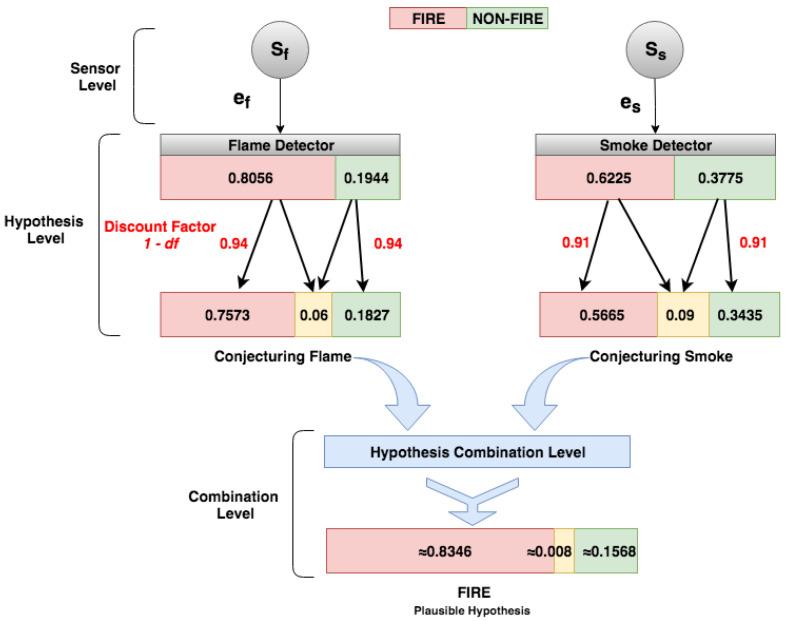
*DST-CEP* computing processing levels of the fire detection system.

Figure 9 shows how IoT application events are processed and calculated in *DST-CEP* computing processing levels. Initially, primitive hypotheses defined in the frame of discernment for this fire alarm application is Θ={Fire,Non-fire}, where Fire denotes that a fire is happening, Non-fire denotes that no fire is happening. All subsets formed by the disjunction of the primitive hypotheses give rise to all possible hypotheses $2^{\Theta }=\{Ø,\left\{Fire\right\},\{Non-fire\},\Theta \}$, where Θ denotes uncertainty about whether there is a fire or non-fire. The mass value of the empty set is zero by definition (Equation (1)).

#### 5.2.1. Sensor Level

In the Sensor Level (Figure 9), two detectors (flame and smoke) send readings (i.e., pieces of evidence) to the *DST-CEP*. Given the formal definition of the event (Section 4.1), we show an instance of the flame event, as follows:

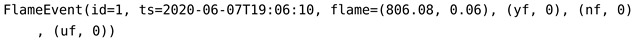


*FlameEvent* is composed of a sensor identification, *ts* is the timestamp, *flame* value is 806.08 nm, discount factor is 0.03, and yf = 0, nf = 0, and uf = 0 are hypotheses “yes fire”, “non-fire” and “uncertain fire” with mass values not calculated at that moment. Consider an example to calculate these values, the flame sensor can detect flames and infrared light sources with wavelengths from 750 nm whose value is the detector threshold. Importantly, we assumed thresholds from sensor manufacturer information related to detection ranges, tests, and sensor sensitivity. Algorithm 1 receives flame data (e.g., 806.08 nm) and checks if the value exceeds the threshold (e.g., 750.0). If yes, then the hypothesis “yes fire” is confirmed (*yf*), the values of *x* and *y* are 0.5 and 1, respectively, and the mass value for this hypothesis should be calculated. The equation of the Algorithm 1 uses the values epl=806.08, thr=750 and Max(epl)=841.74 (the highest value of the last 10 sensor readings from the sliding window). From these values, the results of the mass values are: *yf* = 0.8056, and *nf* = 0.1944.

#### 5.2.2. Hypothesis Level

In the Hypothesis Level, *DST-CEP* rules perform hypothesis conjectures with the associated mass values. The mass values (yf, nf, and uf) should be calculated. For this purpose, the mass function (see Section 4.3.1) is applied based on Algorithm 1. The uncertainty (uf) of the production source of events needs to be considered in the hypothesis conjecture. Therefore, mass values attributed by the flame detector are hypotheses yf=0.8056 and nf=0.1944. By using the mass function with the discount factor 0.06, Equation (6) generates results for *FlameEvent* with mass values for hypotheses yf=0.7573, nf=0.1827, and uf=0.06 (see Figure 9).



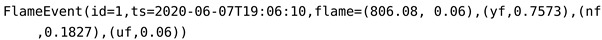



Likewise, all detectors in *DST-CEP* generate hypothesis conjecture, therefore the mass functions from smoke and temperature detectors with $thr_{s}=75.0$ and $thr_{t}=40.0$ generate the following event instances:

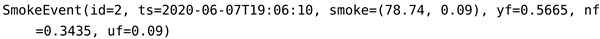


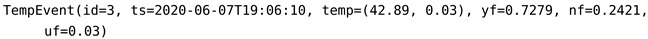


Code 4 shows the CEP rule of the flame detector. Data extracted from the method *FlameEvent()* (line 6) are processed in the mass function calculation (line 4) and inserted in *FlameEvidence* (line 1).

Code 4: CEP rule of flame detector.

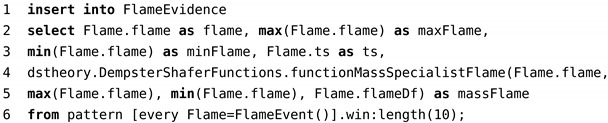



The sliding window (see Code 4, line 6) is continuously formed by the latest ten events. While consuming new events, the sliding window captures flame variations and real-time changes of minimum, maximum, and intervals, which result in different mass values for hypotheses.

#### 5.2.3. Hypothesis Combination Level

**Figure 10:**
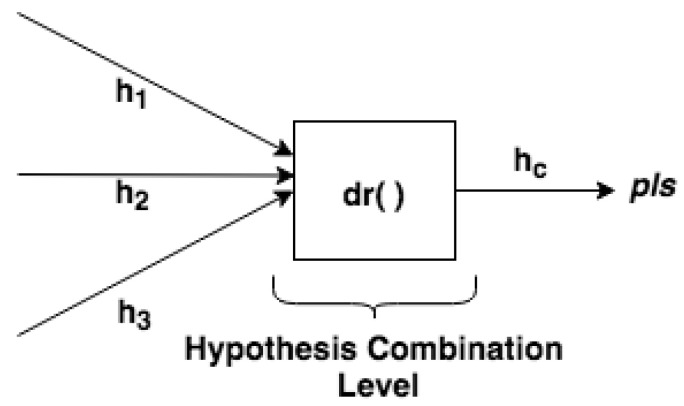
*DST-CEP* Hypothesis Combination Level (depicted from Figure 2).

Finally, in the Hypothesis Combination Level, data collected from various information sources are combined to obtain the most accurate fire identification (Figure 9). In this step, the combination of the hypotheses generated by multiple detectors occurs in the output. There is a result (see Figure 10) of the combined hypotheses ($h_{c}$), in which the most plausible hypothesis (*pls*) can be detected.

From a set of derived hypothesis conjectures, the Dempster combination rule dr() is used (Equations (2)–(4)) to calculate the combination of hypotheses from temperature, smoke, and flame detectors. When three or more detectors contribute information, the Dempster rule application is repeated using the inner elements calculated from the first application of the rule (e.g., the combination of $h_{1}$ with $h_{2}$) with the hypothesis ($h_{3}$) from the next detector. This means that the Dempster combination rule can be generalized from Equation (2) to more than two hypotheses, as presented in Equation (7), and the order of several combinations does not affect the result.

(7) $$m_{1}⊕m_{2}⊕m_{3}⊕\ldots =\left(\left(\left(m_{1}⊕m_{2}\right)⊕m_{3}\right)⊕\ldots \right)$$

The rule used to process the three events (*TempEvent*, *SmokeEvent*, *FlameEvent*) that occur in one minute is presented in Code 5. In lines 3–5, the mass values of hypotheses are captured from the temperature, smoke, and flame detectors. The Dempster combination rule is called in lines 6–9 using the mass values. Lines 10–13 check if the hypothesis conjectures occurred within 1 min.

Code 5: CEP rule of Dempster combination rule.

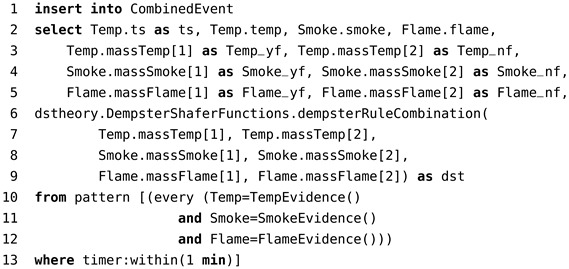



The Dempster rule is calculated with the following mass values from the three events (*TempEvent*, *SmokeEvent*, and *FlameEvent*). Therefore, the mass values contributed by detectors are:

- $m_{Flame}\left(yf\right)=0.7573$, $m_{Flame}\left(nf\right)=0.1827$ and $m_{Flame}\left(uf\right)=0.06$
- $m_{Smoke}\left(yf\right)=0.5665$, $m_{Smoke}\left(nf\right)=0.3435$ and $m_{Smoke}\left(uf\right)=0.09$
- $m_{Temp}\left(yf\right)=0.7279$, $m_{Temp}\left(nf\right)=0.2421$ and $m_{Temp}\left(uf\right)=0.03$

The hypothesis combination $h_{c}$ (see Figure 10) generated by multiple sensors is given by Equations (2)–(4), and the results are $m_{hc}\left(yf\right)$ = 0.9342, $m_{hc}\left(nf\right)$ = 0.0654, and $m_{hc}\left(uf\right)$ = 0.004. From combined hypothesis $h_{c}$, it is possible to detect the most plausible hypothesis, which is *Fire*. The final result presented is based on pieces of evidence from all detectors in favor of the fire hypothesis, i.e., the highest mass value from each detector conjecturing fire hypothesis.

However, it is important to observe contradictions between hypotheses in this case study, i.e., the highest mass value from detectors conflicting in favor of fire and non-fire hypotheses. Table 1 illustrates examples of fire and non-fire conditions and conflicting hypotheses, which are described below.

- Conflict 1: it illustrates a real fire condition of slow combustion, i.e., a high amount of smoke at the beginning (smoke detector notifies fire, $m_{Smoke}\left(yf\right)$ = 0.7022); but with slow heating and the absence of flames, the other two detectors notify non-fire ($m_{Temp}\left(nf\right)$ = 0.4910 and $m_{Flame}\left(nf\right)$ = 0.6589). In this situation, the result of the *DST-CEP* combination ($m_{DST}\left(yf\right)$ = 0.5519) concluded the real condition;
- Conflict 2: the temperature detector shows that the mass value for fire hypothesis is high ($m_{Temp}\left(yf\right)$ = 0.8206), while the evidence collected by smoke detector notify non-fire $m_{Smoke}\left(nf\right)$ = 0.5519. By using *DST-CEP*, the final result of the combination concluded the correct condition ($m_{DST}\left(yf\right)$ = 0.7967);
- Conflict 3: temperature sensors may be close or in contact with heated materials (e.g., electric wires, heated plates); in this case, the temperature detector indicates abnormal, but low, therefore, by mistake, conjecturing the fire hypothesis ($m_{Temp}\left(yf\right)$ = 0.6649), while the other two detectors indicate the absence of fire $m_{Flame}\left(nf\right)$ = 0.6721 and $m_{Smoke}\left(nf\right)$ = 0.5003. *DST-CEP* correctly concludes non-fire ($m_{DST}\left(nf\right)$ = 0.5559);
- Conflict 4: it illustrates a real condition of large amount of flame at the beginning (fast combustion). In this case, the flame detector notifies fire ($m_{Flame}\left(yf\right)$ = 0.5493), while the temperature sensor, in contradiction, indicates non-fire ($m_{Temp}\left(nf\right)$ = 0.5260). However, pieces of evidence considered together by the *DST-CEP* conclude fire ($m_{DST}\left(yf\right)$ = 0.5720), coinciding with the real condition.

As illustrated, the final results represent synthetic effects of all pieces of evidence of hypothesis conjectures. The *DST-CEP* solution achieves positive results even when sensors are in abnormal situations and generate conflicting hypotheses.

## 6. Experimental Evaluation

In this section, we present an investigation on the benefits of uncertainty treatment in IoT applications. In the previous section, we presented a multi-sensor fire outbreak detection system. From that case study, we evaluated the *DST-CEP* solution with a dataset collected from real sensors. Therefore, the objective of this experiment was to evaluate the performance of the *DST-CEP* approach compared to individual detectors, rules considering multiple detectors, and probabilistic models.

### 6.1. Implementation Aspects

Based on the Dempster–Shafer Theory, all formalism presented in our *DST-CEP* solution was implemented with TDS functions using CEP and Java technologies. Our fire detection system uses CEP to store continuous queries executed while data flows over the queries. Statements of queries written in EPL allows expressing rich conditions and correlations between events and sliding windows, thus minimizing the development effort required to configure systems that can react to complex situations. The EPL is provided by Esper and implemented in our solution. Esper is an open source engine for CEP, designed for real-time Event-Driven Architecture (EDA) written in Java [64]. Esper is capable of triggering custom actions written as Plain Old Java Objects (POJO) when event conditions occur between event streams [64,68]. Esper enables to import classes and packages, which allows us to declare methods in these classes accessed within CEP rules. The Esper EPL is a rich declarative language for rule specification, continuous queries, including all SQL operators (SQL-like extended), language constructions for interaction and window definitions, and output generation [68]. Esper EPL statements present expressiveness and concepts that extend capabilities of the SQL.

### 6.2. Fire Outbreak Dataset and Sensor Models

In this experiment, we used a dataset collected by Umoh at al. [69]. The authors graciously provided their dataset with 2100 records from three sensors: DHT11 used as temperature sensor, MQ-2 smoke sensor, and LM393 flame sensor. The experiment conducted in [69] reproduced fire and environment conditions. Therefore, the dataset comprises three features (temperature, smoke, and flame), TS is the timestamp, and a real situation with an output label called “fire outbreak detection” (i.e., the ground truth). The label with value 0 is a negative detection (i.e., “Non-fire”), while 1 is a positive detection (i.e., “Fire”). A sample of this dataset is presented in Table 2.

### 6.3. Metrics

We analyzed the following well-known performance metrics [70]: Accuracy, Precision, Recall, and F-Measure. For this purpose, we represent the results using the matrix confusion as follows:

- true positive (*TP*): when there is a correct conjecture of positive value. The value of fire detection from the dataset is yes fire, and the value of plausible hypothesis from the solution is also yes fire;
- true negative (*TN*): when there is a correct conjecture of negative value. The value of fire detection from the dataset is non-fire, and the value of plausible hypothesis from the solution is also non-fire;
- false positive (*FP*): when there is a wrong conjecture (i.e., contradiction) of negative value. The value of fire detection from the dataset is non-fire, and the value of plausible hypothesis from the solution is yes fire;
- false negative (*FN*): when there is a wrong conjecture of positive value. The value of fire detection from the dataset is yes fire, and the value of plausible hypothesis from the solution is non-fire.

From the above results, we can calculate Accuracy (Acc), Precision (Prec), Recall (Rec), and F-Measure (F1) [70]. We also analyzed Receiver Operation Characteristic (ROC) curves and Area Under Curve (AUC). Within the simplified version of the ROC approach, we can effortlessly display trade-offs between sensitivity and specificity outcomes. The mathematical expressions are presented below.

(8) $$Acc=\frac{TP+TN}{TP+TN+FP+FN}$$

(9) $$F1=\frac{2*(Prec*Rec)}{Prec+Rec}$$

(10) $$Prec=\frac{TP}{TP+FP}$$

(11) $$Sensitivity=\frac{TP}{TP+FN}=Rec$$

(12) $$Specificity=\frac{TN}{FP+TN}=1-\frac{FP}{FP+TN}$$

In terms of fire detectors, the Sensitivity (Equation (11)) can be defined as the ability that a detector has to recognize fire (true positive rate). In comparison, the Specificity (Equation (12)) is defined as the ability that a detector has to recognize non-fire (true negative rate), or the ability to exclude false fire notifications (1−Specificity).

### 6.4. Baseline

The first objective of our experiment is to evaluate the gain of using the *DST-CEP* over standalone CEP for an IoT application. Thus, we measure the performance rates to detect fire and non-fire with *DST-CEP*, when uncertainty is managed, and CEP without considering uncertainty. The second objective of this experiment is to evaluate the performance of the *DST-CEP* approach compared to individual and multiple detectors considering contradictions between hypotheses, i.e., the highest mass value from detectors conflicting in favor of fire and non-fire hypotheses. The third objective of this experiment is to compare results achieved by our approach with those by state-of-the-art models. Therefore, we compared the performance of our solution with results from different information sources, which are presented in Table 3.

The performance of each detector is verified from the mass values and compared with the *DST-CEP* solution. For this purpose, a single detector is deployed in the EPN, and results are evaluated. For example, the temperature detector is considered individually, and the obtained mass values are verified. The mass values generated by the temperature detector are recorded and evaluated in relation to our solution.

Our experiment also explores the processing of CEP rules without uncertainty to compare results with *DST-CEP* rules considering uncertainty. We created rules without uncertainty considering the three detectors, i.e., temperature (TD), smoke (SD), and flame (FD). We defined the same thresholds used in the case study, and then combine the results of the detectors using two operators of Propositional Logic (PL): the conjunction using the operator (∧) and the disjunction (∨).

- Rule of conjunction (RC) written in PL as TD∧SD∧FD uses the conjunction of three results from the detectors TD, SD, and FD.
- Rule of disjunction (RD) written in PL as TD∨SD∨FD uses the disjunction of three results from the detectors.

The response values of the RC and RD attends 16 possibilities of the truth-values from the detectors TD, SD, and FD.

Our experiment also explores probabilistic models based on [19], which admit events characterized by a degree of uncertainty using probability theory. Composite events (i.e., derived events) combine primitive events. When sensor readings come from different and independent sources, then the overall probability of the composite event is the product of the probability of primitive events. The Normal Probabilistic Model (NPM) assumes that the probability distribution function (*pdf*) of the error is known (i.e., measurement errors of sensors have a normal distribution N(0,1)). We improved NPM, now called IPM, by specifying the standard deviation N(0,0.09), N(0,0.06), and N(0,0.03) for smoke, flame, and temperature detectors, respectively. These values correspond to discount factors that express the inaccuracy of the sensors used in our approach. A lower standard deviation corresponds to a more accurate sensor in order to find better results.

### 6.5. Results and Analysis

Table 4 shows the results of performance metrics of our experiment when analyzing the detectors working individually. The temperature detector presents the best performance in *Acc*, *Rec*, and *F1* but low *Prec* (77.33%). The smoke detector also presents low *Prec* (77.98%), and the other metrics with performance below the temperature detector. This fact is why the temperature detector has the largest number of fire notifications (TP) and has the worst number of false fire notifications (FP). The flame detector presents the worst number of non-notified fire occurrences (FN), so the worst performance with the lowest *Rec* (57.30%).

Individual detectors have low overall performance, while the *DST-CEP* solution achieves the best results on all metrics. We consider FNs more important in this fire scenario because it is critical to neglect a real fire occurrence. In this perspective, the *DST-CEP* has the number of FNs significantly reduced compared to individual detectors. This fact is why our approach gives a *Rec* of 97.30%, which shows its ability to detect almost all the fire events correctly. The Recall represents the sensitivity of the *DST-CEP*, in which the true positive rate is not ignored, and the critical false negatives of real fire occurrences are reduced to a few. To evaluate the *DST-CEP* overall performance in the fire detection system, we analyze Accuracy and F-Measure. Our solution reaches *Acc* 95% and *F1* 91.14%, which shows that it detects fire and non-fire events correctly with even less difficulty. *Prec* of 85.71% means that our solution increases by approximately 8% of the best precision between detectors.

**Figure 11:**
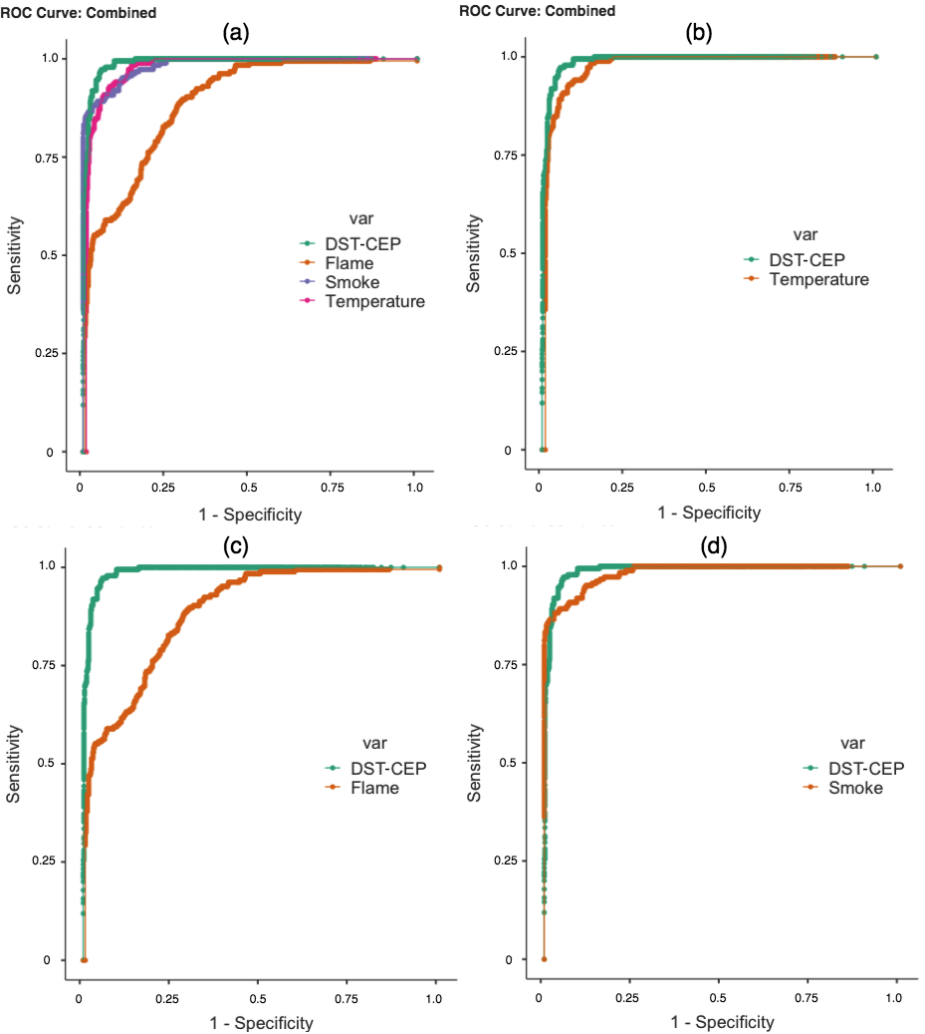
ROC curves analyzed. (**a**) ROC curves of detectors and *DST-CEP*; (**b**) ROC curves of temperature detector and *DST-CEP*; (**c**) ROC curves of flame detector and *DST-CEP*; and (**d**) ROC curves of smoke detector and *DST-CEP*.

Another point of our analysis concerns the ROC curves and AUC. The results of the fire detectors and *DST-CEP* produced answers of mass values for hypotheses. In this case, the relationship in Figure 11 between sensitivity and specificity of quantitative mass values is represented across “cutoff point” values. Mass values less than or equal to the cutoff point is classified as non-fire, and a response greater than the cutoff point is classified as fire.

In practice, it is desirable to have a result that is both sensitive and specific. In Figure 11a, we present the ROC curves that refer to fire detectors and *DST-CEP*. We can identify that the *DST-CEP* curve is the most sensitive and specific. In Figure 11b,c, the result of the *DST-CEP* curve (green line) demonstrates greater discriminating power of sensitivity and specificity, and the flame curve is the worst one. When the *DST-CEP* curve crosses the smoke curve (Figure 11d), more attention is required because there is no dominant relationship between them. AUC is presented as a summary of the ROC curves, and can deal with overlapping curves [71]. As observed in Table 5, the AUC of the *DST-CEP* shows a better performance overcoming all results of individual detectors. A high AUC means that the fire detection has low false positive rates (high specificity) when the sensitivity is high, which is the desired behavior for our *DST-CEP* solution.

It is worth noting that RC, RD, NPM, and IPM do not generate results with continuous values mostly, consequently not enabling a curve projection for ROC analysis.

Table 6 shows the results of our experiment when analyzing performance metrics of rules without uncertainty from RC and RD. The fire notification by RC is precise because it requires fire confirmation in all three detectors. For this reason, RC is capable of recognizing fire occurrences without any false positive, so reaching 100% precision. However, the Recall metric is low (55.68%), and Accuracy and F-Measure values are below our solution. Fire notification by the RD is more sensible to fire notifications because this rule requires fire confirmation from only one detector. For this reason, RD detects fire notifications without any false negative (Recall of 100%). However, RD generates many false positive notifications (i.e., low Precision). Our *DST-CEP* solution overcomes RD in Precision, Accuracy, and F-Measure metrics.

Table 7 shows the results of our experiment when analyzing performance metrics of the probabilistic models. Our approach outperforms the results of these models. The better result of the NPM is accuracy with 81.14%. It is worth noting that all results of NPM do not outperform the rules without uncertainty. This phenomenon is repeated in [19], where rules without considering uncertainty do not have accuracy overcome. We find better results in IPM compared to the NMP. Even with the improvement, *DST-CEP* outperforms the IPM in all performance metrics.

### 6.6. Discussion

#### 6.6.1. What Went Well

We performed an experimental evaluation that allowed us to analyze the performance of the *DST-CEP* solution considering state-of-art metrics in a real-time fire detection application. Results confirmed the gains of our approach. When analyzing the detectors, the *DST-CEP* presented the best performance in Accuracy, Precision, Recall, and F-Measure. We achieved the best ROC curve even when combining information from detectors with low performance causing conflicting fire notifications, and the *DST-CEP* curve had better sensitivity and specificity in this situation. The case study demonstrated that the most plausible hypothesis from the combination of data provided by unreliable sensors was obtained. The AUC values confirmed the promising result of the *DST-CEP*.

Additionally, the *DST-CEP* demonstrated significant improvements in performance results over standalone CEP without considering uncertainty (i.e., the rules RC and RD), which attends the exhaustive 16 possibilities of combining the results from detectors. RC had the best Precision, but it had Recall, Accuracy, and F-Measure results below our solution. RD was more sensible to fire notifications and had the best Recall. However, RD generated many false positive notifications, thus our solution gained in Precision, Accuracy, and F-Measure results.

Eventually, an acceptance threshold for events can be used in our approach. This situation is useful in IoT applications where resulting events are sufficiently critical to accept only notifications with high reliability. For example, when accepting non-fire notifications only with values above 0.9, thus decreasing false non-fire notifications, then avoiding false negatives, which is critical in this scenario. This is a feature that *DST-CEP* can provide, and standalone CEP without considering uncertainty cannot.

*DST-CEP* demonstrated to be suitable and flexible to deal with the uncertainty issues described in this study. Several related works are based on the probability theory [6,7,13,19,37,42]. In those approaches, the role of sample space (Ω), which is the set of all possible outcomes, resembles the frame of discernment (Θ) in *DST-CEP*. However, the difference is the number of possible hypotheses, which is $\Theta^{2}$ in our approach. This fact implies, for example, that a sample space would typically hold either yes fire (yf) or non-fire (nf). However, the uncertain fire (uf) is not modeled in a sample space, i.e., uncertain observations cannot be modeled. In our study, we aimed at supporting multiple interpretations of one data from a frame of discernment, attending uncertainty about all possibles hypotheses and any combination of them. Therefore, *DST-CEP* offers an alternative to probabilistic approaches for representing and reasoning over uncertain information.

We performed experiments with probabilistic models based on [19]. In such models, parameters are manually estimated or calculated by domain specialists. These users are expected to extend BNs (i.e., BN enrichment) to capture a-priori knowledge about aspects of the environment that cannot be observed by information sources. During the enrichment, the BN can be edited by changing the a-priori distribution of probability values. In *DST-CEP*, we dealt with uncertainty in events by explicitly assigning them with uncertain information from real sensors in a formal representation. This permits associating a level of uncertainty related to the event source (e.g., sensor readings) in a natural way without requiring any user computation. We also dealt with propagation of uncertainty to rules using the Dempster combination rule, which presents positive synthetic effects of all pieces of evidence of hypothesis conjectures even when sensors are in abnormal situations. Unlike probabilistic approaches, which use simulated datasets, we used a dataset composed of data from real sensors, and *DST-CEP* obtained significant performance results in relation to the studied probabilistic models.

#### 6.6.2. Identified Limitations

From the development of our proposed solution and experimental evaluation, we were able to identify some limitations. A difficulty with the *DST-CEP* approach is to model mass functions. It is worth noting that the Dempster–Shafer Theory does not focus on the judgment, calculation, or mechanism by which the mass value is determined. This theory focuses on combining distinct mass values based on pieces of evidence from multiple sources. Therefore, modeling mass functions may be complex depending on the problem domain or application scenario.

Another difficulty is the absence of consensus on selecting the optimal threshold for a detector rule to generate a notification. There is scant guidance on the selection of thresholds at which detectors are required to alarm. Specifically in the case study presented, in fire detection systems, the nature of fire is a science apart. For example, many variables such as fuel type, detector design, burn mode, and smoke aging, can affect the fire detector response [72]. We have assumed thresholds from the sensor manufacturer information.

## 7. Conclusions and Future Works

The objective of the proposed study was to investigate the identification and treatment of uncertainty in events and processing rules from multi-sensor data in CEP-based IoT applications. We first developed the *DST-CEP* approach to adequately model and deal with uncertainty in sensor data and its propagation to CEP rules. This approach is based on the Dempster–Shafer Theory. An instance of our architectural model was employed in a case study of IoT application, and DST elements were used in several stages of our architectural model. Specifically, we presented a scenario demonstrating the applicability of the proposed solution in a multi-sensor fire outbreak detection system to detect fire alarms from sensors in uncertainty situations.

Future plans include an investigation on the selection of optimal thresholds for detectors in an automated and standardized way. We also plan to explore and evaluate other application scenarios in which *DST-CEP* can be applied. We would also like to use other DST elements such as the belief interval.

## Figures and Tables

**Table 1:** Results of conflicting hypotheses and *DST-CEP*.

Sources	Conflict 1		Conflict 2		Conflict 3		Conflict 4	
	Fire	Non-Fire	Fire	Non-Fire	Fire	Non-Fire	Fire	Non-Fire
Temperature Detector	0.4790	0.4910	0.8206	0.1494	0.6649	0.3051	0.4440	0.5260
Smoke Detector	0.7022	0.2078	0.4882	0.4218	0.4097	0.5003	0.4956	0.4144
Flame Detector	0.2811	0.6589	0.3881	0.5519	0.2679	0.6721	0.5493	0.3907
Real Condition	Fire		Fire		Non-fire		Fire	
*DST-CEP*	0.5519	0.4474	0.7967	0.2027	0.4399	0.5559	0.5720	0.4274

**Table 2:** A sample of the fire outbreak dataset.

TS	Temp	Smoke	Flame	Label
1519785480	33.566	65.797	483.05	0
1519785510	37.682	66.021	516.66	0
1519785540	45.647	79.257	920.62	1

**Table 3:** Baseline of comparison.

Sources	Description
Temperature Detector (TD)	Performance of the temperature detector to conjecture fire and non-fire.
Smoke Detector (SD)	Performance of the smoke detector to conjecture fire and non-fire.
Flame Detector (FD)	Performance of the flame detector to conjecture fire and non-fire.
Rule of Conjunction (RC)	Performance of the rule of conjunction without uncertain to conjecture fire and non-fire.
Rule of Disjunction (RD)	Performance of the rule of disjunction without uncertain to conjecture fire and non-fire.
Normal Probabilistic Model (NPM)	Performance of the probabilistic model where each event notification is accompanied by a probability of occurrence. The probability distribution function of the error is known N(0,1).
Improved Probabilistic Model (IPM)	Performance of the probabilistic model where each event notification is accompanied by a probability of occurrence. The probability distribution function of the error is known with accuracy improved.

**Table 4:** Results of performance metrics from individual detectors.

Sources	Performance Metrics			
	Accuracy	Precision	Recall	F-Measure
TD	91.14%	77.33%	94.05%	84.88%
SD	91.00%	77.98%	91.89%	84.37%
FD	84.14%	76.81%	57.30%	65.63%
*DST-CEP*	95.00%	85.71%	97.30%	91.14%

**Table 5:** AUC results.

Area Under Curve			
*DST-CEP*	SD	TD	FD
0.99032	0.98368	0.97528	0.88799

**Table 6:** Results of performance metrics of conjunction and disjunction rules without uncertainty.

Sources	Performance Metrics			
	Accuracy	Precision	Recall	F-Measure
RC	88.29%	100.0%	55.68%	71.53%
RD	93.14%	79.40%	100.0%	88.52%
*DST-CEP*	95.00%	85.71%	97.30%	91.14%

**Table 7:** Results of performance metrics from the probabilistic model.

Sources	Performance Metrics			
	Accuracy	Precision	Recall	F-Measure
NPM	81.14%	67.32%	55.68%	60.95%
IPM	81.43%	68.21%	55.68%	61.31%
*DST-CEP*	95.00%	85.71%	97.30%	91.14%

## Abbreviations

The following abbreviations are used in this manuscript:

AUC	Area Under Curve
BN	Bayesian Network
CEP	Complex Event Processing
CEP2U	Complex Event Processing under Uncertainty
DST	Dempster–Shafer Theory
DSTBB	*DST-CEP* Building Blocks
EDA	Event-Driven Architecture
EPA	Event Processing Agent
EPL	Event Processing Language
EPN	Event Processing Network
FD	Flame Detector
FoD	Frame of Discernment
FSCEP	Fuzzy Semantic Complex Event Processing
IPM	Improved Probabilistic Model
IoT	Internet of Things
NPM	Normal Probabilistic Model
POJO	Plain Old Java Objects
RC	Rule of Conjunction
RD	Rule of Disjunction
RDF	Resource Description Framework
ROC	Receiver Operation Characteristic
SD	Smoke Detector
SQL	Structured Query Language
TD	Temperature Detector

## References

[1] Poli J.P., Boudet L. (2018). A fuzzy expert system architecture for data and event stream processing. Fuzzy Sets Syst..

[2] Miorandi D., Sicari S., De Pellegrini F., Chlamtac I. (2012). Internet of Things: Vision, Applications and Research Challenges. Ad Hoc Netw..

[3] Gubbi J., Buyya R., Marusic S., Palaniswami M. (2013). Internet of Things (IoT): A Vision, Architectural Elements, and Future Directions. Future Gener. Comput. Syst..

[4] Abad F.A.T., Caccamo M., Robbins B. A fault resilient architecture for distributed cyber-physical systems. Proceedings of the 2012 IEEE 18th International Conference on Embedded and Real-Time Computing Systems and Applications (RTCSA).

[5] Luckham D., Schulte W.R. (2012). Glossary of Terminology: The Event Processing Technical Society: (EPTS) Glossary of Terms—Version 2.0. Event Processing for Business.

[6] Wasserkrug S., Gal A., Etzion O. (2012). A Model for Reasoning with Uncertain Rules in Event Composition Systems. arXiv.

[7] Wasserkrug S., Gal A., Etzion O., Turchin Y. (2012). Efficient processing of uncertain events in rule-based systems. IEEE Trans. Knowl. Data Eng..

[8] Flouris I., Giatrakos N., Deligiannakis A., Garofalakis M., Kamp M., Mock M. (2017). Issues in complex event processing: Status and prospects in the Big Data era. J. Syst. Softw..

[9] Anicic D., Rudolph S., Fodor P., Stojanovic N. (2012). Stream Reasoning and Complex Event Processing in ETALIS. Semant. Web.

[10] Chandy M.K., Etzion O., von Ammon R. (2011). Executive Summary and Manifesto—Event Processing. Dagstuhl Seminar Proceedings.

[11] Teymourian K. (2014). A Framework for Knowledge-Based Complex Event Processing. Ph.D. Thesis.

[12] Wasserkrug S., Gal A., Etzion O., Turchin Y. Complex event processing over uncertain data. Proceedings of the Second International Conference on Distributed Event-Based Systems.

[13] Artikis A., Etzion O., Feldman Z., Fournier F. Event processing under uncertainty. Proceedings of the 6th ACM International Conference on Distributed Event-Based Systems.

[14] Wasserkrug S., Gal A., Etzion O. A taxonomy and representation of sources of uncertainty in active systems. Proceedings of the International Workshop on Next Generation Information Technologies and Systems.

[15] Akila V., Govindasamy V., Sandosh S. Complex event processing over uncertain events: Techniques, challenges, and future directions. Proceedings of the 2016 International Conference on Computation of Power, Energy Information and Commuincation (ICCPEIC).

[16] Luckham D.C. (2001). The Power of Events: An Introduction to Complex Event Processing in Distributed Enterprise Systems.

[17] Ye J., Dobson S., McKeever S. (2012). Situation identification techniques in pervasive computing: A review. Pervasive Mob. Comput..

[18] Alevizos E., Skarlatidis A., Artikis A., Paliouras G. (2015). Complex event recognition under uncertainty: A short survey. EDBT/ICDT 2015 Workshops.

[19] Cugola G., Margara A., Matteucci M., Tamburrelli G. (2015). Introducing uncertainty in complex event processing: Model, implementation, and validation. Computing.

[20] Shen Z., Kawashima H., Kitagawa H. Lineage-based Probabilistic Event Stream Processing. Proceedings of the 2008 Ninth International Conference on Mobile Data Management Workshops, MDMW.

[21] Zhang H., Diao Y., Immerman N. (2013). Recognizing patterns in streams with imprecise timestamps. Inf. Syst..

[22] Ré C., Letchner J., Balazinksa M., Suciu D. Event queries on correlated probabilistic streams. Proceedings of the 2008 ACM SIGMOD International Conference on Management of Data.

[23] Chuanfei X., Shukuan L., Lei W., Jianzhong Q. Complex event detection in probabilistic stream. Proceedings of the Web Conference (APWEB), 2010 12th International Asia-Pacific.

[24] Wang Y., Cao K., Zhang X. (2013). Complex event processing over distributed probabilistic event streams. Comput. Math. Appl..

[25] Li Z., Ge T., Chen C.X. *ε*-matching: Event processing over noisy sequences in real time. Proceedings of the 2013 ACM SIGMOD International Conference on Management of Data.

[26] Stowell D., Plumbley M.D. (2012). Segregating event streams and noise with a Markov renewal process model. arXiv.

[27] Agrawal J., Diao Y., Gyllstrom D., Immerman N. Efficient pattern matching over event streams. Proceedings of the 2008 ACM SIGMOD International conference on Management of Data.

[28] Dempster A. (1967). Upper and Lower Probabilities Induced by a Multivalued Mapping. Ann. Math. Stat..

[29] Shafer G. (1976). A Mathematical Theory of Evidence.

[30] Etzion O., Niblett P. (2010). Event Processing in Action.

[31] Stühmer R. (2015). Web-Oriented Event Processing.

[32] Chandy K.M., Schulte W.R. (2010). Event Processing—Designing IT Systems for Agile Companies.

[33] Roriz Junior M. (2017). DG2CEP: An On-Line Algorithm for Real-Time Detection of Spatial Clusters from Large Data Streams through Complex Event Processing. Ph.D. Thesis.

[34] Rakowsky U.K. (2007). Fundamentals of the Dempster-Shafer theory and its applications to reliability modeling. Int. J. Reliab. Qual. Saf. Eng..

[35] Stephens W.N. (1968). Hypotheses and Evidence.

[36] Alevizos E., Skarlatidis A., Artikis A., Paliouras G. (2017). Probabilistic Complex Event Recognition. ACM Comput. Surv..

[37] Wasserkrug S., Gal A., Etzion O. (2005). A Model for Reasoning with Uncertain Rules in Event Composition Systems. Proceedings of the Twenty-First Conference on Uncertainty in Artificial Intelligence.

[38] Artikis A., Sergot M., Paliouras G. A logic programming approach to activity recognition. Proceedings of the 2nd ACM International Workshop on Events in Multimedia.

[39] Artikis A., Skarlatidis A., Portet F., Paliouras G. (2012). Logic-based event recognition. Knowl. Eng. Rev..

[40] Jarraya A., Ramoly N., Bouzeghoub A., Arour K., Borgi A., Finance B. (2016). A fuzzy semantic CEP model for situation identification in smart homes. ECAI 2016: 22nd European Conference on Artificial Intelligence.

[41] Rincé R., Kervarc R., Leray P. Complex Event Processing Under Uncertainty Using Markov Chains, Constraints, and Sampling. Proceedings of the International Joint Conference on Rules and Reasoning.

[42] Ma J., Liu W., Miller P. Event modelling and reasoning with uncertain information for distributed sensor networks. Proceedings of the International Conference on Scalable Uncertainty Management.

[43] Ma J., Liu W., Miller P., Yan W. Event composition with imperfect information for bus surveillance. Proceedings of the 2009 Sixth IEEE International Conference on Advanced Video and Signal Based Surveillance.

[44] Ma J., Liu W., Miller P. (2012). An evidential improvement for gender profiling. Belief Functions: Theory and Applications.

[45] Ma J., Liu W., Miller P., Zhou H. (2016). An evidential fusion approach for gender profiling. Inf. Sci..

[46] Moreno N., Bertoa M.F., Burgueño L., Vallecillo A. (2019). Managing measurement and occurrence uncertainty in complex event processing systems. IEEE Access.

[47] Xiao F. (2017). A novel evidence theory and fuzzy preference approach-based multi-sensor data fusion technique for fault diagnosis. Sensors.

[48] Denoeux T. (1995). A k-nearest neighbor classification rule based on Dempster-Shafer theory. IEEE Trans. Syst. Man Cybern..

[49] Liu Z.G., Pan Q., Dezert J., Martin A. (2016). Adaptive imputation of missing values for incomplete pattern classification. Pattern Recognit..

[50] Fu C., Xu D.L. (2016). Determining attribute weights to improve solution reliability and its application to selecting leading industries. Ann. Oper. Res..

[51] Deng X., Jiang W. (2018). An evidential axiomatic design approach for decision making using the evaluation of belief structure satisfaction to uncertain target values. Int. J. Intell. Syst..

[52] Jiang W., Wang S., Liu X., Zheng H., Wei B. (2017). Evidence conflict measure based on OWA operator in open world. PLoS ONE.

[53] Jiang W., Wang S. (2017). An Uncertainty Measure for Interval-valued Evidences. Int. J. Comput. Commun. Control.

[54] Zhang X., Deng Y., Chan F.T., Adamatzky A., Mahadevan S. (2016). Supplier selection based on evidence theory and analytic network process. Proc. Inst. Mech. Eng. Part B J. Eng. Manuf..

[55] Liu T., Deng Y., Chan F. (2018). Evidential supplier selection based on DEMATEL and game theory. Int. J. Fuzzy Syst..

[56] Kang B., Chhipi-Shrestha G., Deng Y., Mori J., Hewage K., Sadiq R. (2018). Development of a predictive model for Clostridium difficile infection incidence in hospitals using Gaussian mixture model and Dempster–Shafer theory. Stoch. Environ. Res. Risk Assess..

[57] Dutta P. (2015). Uncertainty modeling in risk assessment based on Dempster–Shafer theory of evidence with generalized fuzzy focal elements. Fuzzy Inf. Eng..

[58] Zhang L., Ding L., Wu X., Skibniewski M.J. (2017). An improved Dempster–Shafer approach to construction safety risk perception. Knowl. Based Syst..

[59] Yuan K., Xiao F., Fei L., Kang B., Deng Y. (2016). Modeling sensor reliability in fault diagnosis based on evidence theory. Sensors.

[60] Jiang W., Xie C., Zhuang M., Tang Y. (2017). Failure mode and effects analysis based on a novel fuzzy evidential method. Appl. Soft Comput..

[61] Perry D.E., Wolf A.L. (1992). Foundations for the study of software architecture. ACM SIGSOFT Softw. Eng. Notes.

[62] Babar M., Arif F. (2017). Smart urban planning using Big Data analytics to contend with the interoperability in Internet of Things. Future Gener. Comput. Syst..

[63] Iorshase A., Caleb S.F. (2016). A neural based experimental fire-outbreak detection system for urban centres. J. Softw. Eng. Appl..

[64] EsperTech (2021). Esper Reference.

[65] Yager R.R., Liu L. (2008). Classic Works of the Dempster-Shafer Theory of Belief Functions.

[66] Liu W., Hong J., McTear M.F., Hughes J.G. (1993). An extended framework for evidential reasoning systems. Int. J. Pattern Recognit. Artif. Intell..

[67] Lowrance J.D., Garvey T.D., Strat T.M. (2008). A framework for evidential-reasoning systems. Classic Works of the Dempster-Shafer Theory of Belief Functions.

[68] Gianpaolo Cugola A.M. (2012). Processing Flows of Information: From Data Stream to Complex Event Processing. ACM Comput. Surv..

[69] Umoh U., Udo E., Emmanuel N. (2019). Support Vector Machine-Based Fire Outbreak Detection System. Int. J. Soft Comput. Artif. Intell. Appl..

[70] Sokolova M., Lapalme G. (2009). A systematic analysis of performance measures for classification tasks. Inf. Process. Manag..

[71] Zhang X., Li X., Feng Y., Liu Z. (2015). The use of ROC and AUC in the validation of objective image fusion evaluation metrics. Signal Process..

[72] Geiman J., Gottuk D.T. (2003). Alarm thresholds for smoke detector modeling. Fire Saf. Sci..

